# Clinical features of ESBL-producing E. coli responsible for bloodstream infections in French patients and molecular characterization of isolates

**DOI:** 10.1186/2047-2994-4-S1-P125

**Published:** 2015-06-16

**Authors:** A Gaultier, N Girard, X Bertrand, R Quentin, NL Van Der Mee-Marquet

**Affiliations:** 1 Département de Bacteriologie et Hygiène, France; 2 Centre Hospitalier Universitaire de Tours, Tours, France; 3 RHC-arlin, Besançon, France; 4 Service d'hygiène, Centre Hospitalier Universitaire de Besançon, Besançon, France; 5 Service de Bactériologie et Hygiène, Centre Hospitalier Universitaire de Tours, Tours, France; 6 Departement de Bactériologie et Hygiène, Réseau des hygiénistes du Centre, Centre Hospitalier Universitaire de Tours, Tours, France

## Introduction

We conducted an annually bloodstream infection (BSI) survey into hospitals overlapping the Centre France region (2.6 million). Since 2005, the incidence of BSIs associated with ESBL-producing *E. coli* (ESBLEc) increased.

## Objectives

To improve the understanding of the pathway and the determination of the risk factors of ESBLEc-BSIs.

## Methods

For each BSI, were reported patient age, sex, recent hospitalization, living in nursing home, recent antibiotherapy, urinary catheterization, BSI source, death within 7days of diagnosis.

BSI isolates were studied: antimicrobial susceptibility, determination of molecular mechanism associated with ESBL-production, genetic diversity of ESBLEc (MLST).

## Results

During the survey (474,953 PDs), 443 *E. coli* BSI were identified, including 31 ESBLEc (7.0%; 30/31 CTX-M). Incidence of community acquired(CA)- and healthcare associated(HCA)-BSI were 0.47/100,000 and 0.040/1,000 PDs, respectively.

## Major findings

For ESBLEc-CA-BSIs, male/female ratio was 1.4, median age 80, urinary BSI source in 50% of cases, recent antibiotherapy in 33 %. Most ESBLEc were resistant to fluoroquinolones (67%), SXT/TMP (67%). High genetic diversity (8 STs including 4 ST131).

For ESBLEc-HCA-BSI, male/female ratio was 0.9, median age 75, urinary BSI source in 63% of cases (recent catheterization in 1/2), recent antibiotherapy in 58%. Most ESBLEC were resistant to fluoroquinolones (79%), SXT/TMP (63%). Low genetic diversity (9 STs including 7 ST131).

Among BSI, ESBLEc-BSI were associated with healthcare (p=0.004), long-stay unit (p=0.018), recent antibiotherapy (p=0.002). ESBLEc were associated with resistance to fluoroquinolones, SXT/TMP and genta./tobramycine (p<0.001).

Among ESBLEc-BSI, clinical determinants and BSI characteristics similar whatever the clonal group excepted for ST131 associated with long-stay unit (p=0.042).

Among ST131-BSI, clinical determinants and BSI characteristics similar for ESBLEc and non ESBLEc excepted median age higher in ESBLEc (80/67).

## Conclusion

Recent antibiotherapy (and easy spread into long-stay units for ST131): likely the major risk factor for ESBLEc BSI.

## Disclosure of interest

None declared.

